# Efficacy and tolerability of granulocyte colony-stimulating factors in cancer patients after chemotherapy: A systematic review and Bayesian network meta-analysis

**DOI:** 10.1038/s41598-019-51982-4

**Published:** 2019-10-25

**Authors:** Yong Wang, Lin Chen, Fen Liu, Ning Zhao, Liyao Xu, Biqi Fu, Yong Li

**Affiliations:** 1 Department of Medical Oncology, The First Affiliated Hospital of Nanchang University, 17 Yongwai Zheng Road, Nanchang, 330000 China; 2 Department of Medical Oncology, The Affiliated Ganzhou Hospital of Nanchang University (Ganzhou People’s Hospital), 18 Meiguan Road, Ganzhou, 341000 China; 3 Department of Internal Neurology, The Affiliated Ganzhou Hospital of Nanchang University (Ganzhou People’s Hospital), 18 Meiguan Road, Ganzhou, 341000 China; 4 Critical Care Medicine, The First Affiliated Hospital of Nanchang University, 17 Yongwai Zheng Road, Nanchang, 330000 China; 5 Department of paediatrics, Children’s Hospital, Zhejiang University School of Medicine, 57 Zugan Road, Hangzhou, 310000 China; 6 Department of Rheumatology, The First Affiliated Hospital of Nanchang University, 17 Yongwai Zheng Road, Nanchang, 330000 China

**Keywords:** Drug development, Cancer

## Abstract

The optimum granulocyte colony-stimulating factor (G-CSF) treatment for cancer patients after being treated with cytotoxic chemotherapy remains unknown. Therefore, a systematic review and Bayesian network meta-analysis were performed to assess the efficacy and tolerability of 11 G-CSF drugs on patients after chemotherapy. A total of 73 randomized controlled trials (RCTs) containing 15,124 cancer patients were included for the final network meta-analysis. Compared with pegfilgrastim, there were a higher risk with filgrastim for incidence of febrile neutropenia (FN) (OR [95% CI]: 1.63 [1.07, 2.46]), and a higher risk with short-acting G-CSF (S-G-CSF) biosimilar and lenograstim for incidence of bone pain (BP) (OR [95% CI]: 6.45 [1.10, 65.73], 5.12 [1.14, 26.12], respectively). Mecapegfilgrastim, lipegfilgrastim and balugrastim were best G-CSF drugs in reducing FN (cumulative probabilities: 58%, 15%, 11%, respectively). S-G-CSF biosimilar, empegfilgrastim, and long-acting G-CSF (L-G-CSF) biosimilar were best G-CSF drugs in reducing severe neutropenia (SN) (cumulative probabilities: 21%, 20%, 15%, respectively). Mecapegfilgrastim, balugrastim, lipegfilgrastim and L-G-CSF biosimilar were best G-CSF drugs in reducing BP (cumulative probabilities: 20%, 14%, 8%, 8%, respectively). Mecapegfilgrastim, lipegfilgrastim and balugrastim might be the most appreciate G-CSF drugs with both good efficacy and tolerability when treating cancer patients after cytotoxic chemotherapy.

## Introduction

Febrile neutropenia (FN) and severe neutropenia (SN) are the most common and serious complications of cancer patients after treatment with cytotoxic chemotherapy^1^. These complications lead to chemotherapy delay, dose reduction, and increased risk of infection^2^. Patients with these complications need to be treated with antibiotics and hospitalization^3^, which indirectly increases the cost for care of these patients^4^. Furthermore, the condition could deteriorate and lead to death as a result of FN and/or SN after chemotherapy^4,5^.

Granulocyte colony-stimulating factors (G-CSFs) promote the growth of neutrophils, decrease the incidence of FN and SN, shorten the time of hospital stay, reduce the severity and duration of neutropenia, decrease the risk of infection, and improve the tolerance to cytotoxic chemotherapy^6^. The guidelines of National Comprehensive Cancer Network (NCCN) recommend primary prophylaxis with G-CSF when the risk of FN associated with chemotherapy regimen is greater than 20%^7^. Filgrastim was the first short acting G-CSF drug approved for treatment of neutropenia by the United States Food and Drug Administration (FDA) in 1991. Subsequently, a number of new G-CSF drugs have been invented for the treatment of neutropenia worldwide. Long-acting G-CSFs (L-G-CSFs) are PEGylated forms of short-acting G-CSFs (S-G-CSFs) with decreased elimination and increased half-life in serum after subcutaneous injection. Moreover, some of these new G-CSF biosimilar drugs are not as glycosylated as filgrastim^8^. Since the structure and mechanism of drugs differ, the effect of different G-CSFs remains unclear.

Bone pain (BP) is the most frequent adverse event associated with G-CSF drugs^6^. Patients might give up treatment due to severe adverse events. The incidence and degree of bone pain after the injection of different G-CSF drugs are diverse^9^. Although some reviews on the difference of several G-CSF drugs have been reported^10,11^, these reviews did not include sufficient studies and samples, trials that assessed new G-CSF drugs, or a complete list of G-CSF drugs. The effect of G-CSFs and the optimum choice remains unclear.

Since there is no evidence from head-to-head trials, pairwise meta-analysis for mixed treatment comparisons between multiple medical interventions appears to be impossible. The Bayesian network meta-analysis, which combined direct and indirect evidence to obtain an estimated effect value, has been considered to be a statistical method for mixed multiple trial data comparisons, when a head-to-head trial is not available^12^. In the present study, a Bayesian network meta-analysis was performed to compare the major 11 G-CSF drugs (balugrastim, empegfilgrastim, filgrastim, S-G-CSF Biosimilar, L-G-CSF Biosimilar, lenograstim, leridistim, lipegfilgrastim, mecapegfilgrastim, pegfilgrastim, and pegteograstim) in terms of efficacy (FN and SN) and tolerability (BP) in the treatment of patients after cytotoxic chemotherapy. This aimed to summarize the direct evidence obtained from the results of randomized controlled trials (RCTs), in order to provide reliable information for guiding clinical treatment decisions.

## Results

### Inclusion studies

**Figure 1:**
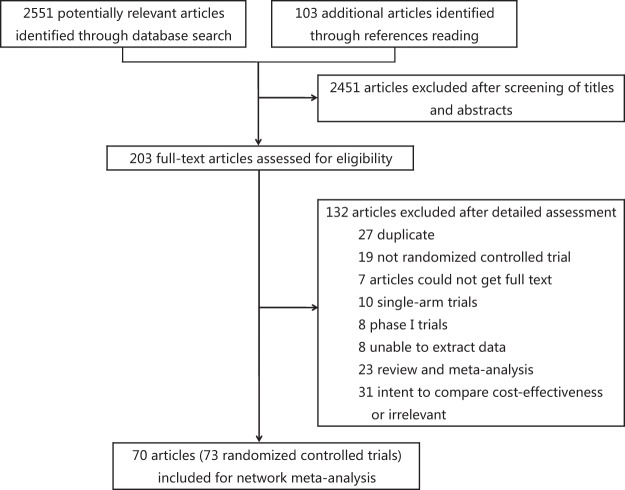
Flowchart of the meta-analysis.

A total of 2,551 potentially relevant articles were identified based on the selection criteria (Fig. 1). After the titles and abstracts were examined, 2,451 literatures that did not meet the criteria were excluded. The full texts of 203 eligible articles were further assessed in detail, and 132 of these were further excluded (Fig. 1). Overall, 70 studies^13–82^ of the 73 RCTs from 1991 to 2018 were included for the final network meta-analysis (Table 1). The assessment of risk of bias indicated low risk of bias among the RCTs (Supplementary Figs S1 and S2). These trials were carried out in 19 countries, and almost half of these clinical trials were conducted in Europe. These trials contained a total of 15,124 cancer patients with 12 kinds of tumors. These 12 types of cancers were breast cancer (BC), lung cancer (LC), gastric cancer (GC), ovarian cancer (OC), head and neck cancer (HNC), colorectal cancer (CRC), germ cell malignancy (GCM), acute myeloid leukemia (AML), acute lymphoblastic leukemia (ALL), lymphoma, sarcoma, and neuroblastoma. These patients were randomly assigned to one of the 12 treatments (11 G-CSF drugs and one placebo group). BC (approximately 42%) was the main disease with the most patients among all kinds of tumors. The additional basic characteristics of all the included studies are presented in Table 1.

**Table 1:** Baseline characteristics of the included studies.

No.	Author. Year	Study design	Country	Tumour type	Stages	Patients	Sex (M/F)	Treatment group	Intervention Dose
1	Crawford *et al*.^13^	Phase III, DB	USA	SCLC	Limited/Extensive	231	149/82	Filgrastim vs. Placebo	5 μg/kg/day vs. -
2	Fosså *et al*.^14^	Phase III, NA	UK	GCM	IV	259	NA	Filgrastim vs. Placebo	5 μg/kg/day vs. -
3	Dunlop *et al*. 1998 study1^15^	NA, NA	UK	HL	I/ II/ III/ IV	25	15/10	Filgrastim vs. Placebo	5 μg/kg/day vs. -
4	Dunlop *et al*. 1998 study2^15^	NA, NA	UK	HL	I/ II/ III/ IV	22	17/7	Filgrastim vs. Placebo	5 μg/kg/day vs. -
5	Geissler *et al*.^16^	Phase III, NA	Australia	ALL	I/ II/ III/ IV	51	27/24	Filgrastim vs. Placebo	5 μg/kg/day vs. -
6	Pinter *et al*.^17^	Phase III, DB	USA	CRC	Advanced	845	512/333	Pegfilgrastim vs. Placebo	6 mg/cycle vs. -
7	Kubo *et al*.^18^	Phase III, DB	Japan	Lymphoma	I/ II/ III/ IV	107	66/41	Pegfilgrastim vs. Filgrastim	3.6 mg/cycle vs. 50 μg/m^2^/day
8	Zhang *et al*.^19^	Phase II, OL	China	BC	NA	86	0/86	Pegfilgrastim vs. Filgrastim	100 μg/kg/cycle vs. 5 μg/kg/day
9	Kosaka *et al*.^20^	Phase III, DB	Japan	BC	I/ II/ III	346	0/346	Pegfilgrastim vs. Placebo	6 mg/cycle vs. -
10	Shi *et al*.^21^	Phase III, OL	China	BC/NSCLC/NHL/HNC	I/ II/ III/ IV	326	128/198	Pegfilgrastim vs. Filgrastim	100 μg/kg/cycle vs. 5 μg/kg/day
11	Hecht *et al*.^22^	Phase II, DB	USA	CRC	II/ III/ IV	241	162/79	Pegfilgrastim vs. Placebo	6 mg/cycle vs. -
12	Fox *et al*.^23^	NA, NA	USA	Sarcomas	III/ IV	34	17/17	Pegfilgrastim vs. Filgrastim	100 μg/kg/cycle vs. 5 μg/kg/day
13	Sierra *et al*.^24^	Phase II, DB	Spain	AML	NA	83	39/44	Pegfilgrastim vs. Filgrastim	6 mg/cycle vs. 5 μg/kg/day
14	Vogel *et al*.^25^	Phase III, DB	USA	BC	I/ II/ III/ IV	928	6/922	Pegfilgrastim vs. Placebo	6 mg/cycle vs. -
15	Grigg *et al*.^26^	Phase II, OL	USA	NHL	I/ II/ III/ IV	27	14/13	Pegfilgrastim vs. Filgrastim	100 μg/kg/cycle vs. 5 μg/kg/day
16	Vose *et al*.^27^	Phase II, OL	USA	Lymphoma	I/ II/ III/ IV	60	36/24	Pegfilgrastim vs. Filgrastim	100 μg/kg/cycle vs. 5 μg/kg/day
17	Green *et al*.^28^	Phase III, DB	Australia	BC	II/ III/ IV	152	1/151	Pegfilgrastim vs. Filgrastim	6 mg/cycle vs. 5 μg/kg/day
18	Holmes *et al*. study1^29^	Phase III, DB	USA	BC	II/ III/ IV	296	3/293	Pegfilgrastim vs. Filgrastim	100 μg/kg/cycle vs. 5 μg/kg/day
19	Holmes *et al*. study2^30^	Phase II, DB	USA	BC	II/ III/ IV	71	0/71	Pegfilgrastim vs. Filgrastim	100 μg/kg/cycle vs. 5 μg/kg/day
20	Zhou *et al*.^31^	Phase III, DB	China	NSCLC	IIIB/IV	151	101/44	Mecapegfilgrastim vs. Placebo	6 mg or 100 μg/kg/cycle vs. -
21	Volovat *et al*.^32^	Phase III, DB	Romania	NSCLC	IIIB/IV	365	325/50	Lipegfilgrastim vs. Placebo	6 mg/cycle vs. -
22	Buchner *et al*.^33^	Phase II, DB	Germany	BC	II/ III/ IV	104	1/103	Lipegfilgrastim vs. Pegfilgrastim	6/mg/cycle vs. 6 mg/cycle
23	Bondarenko *et al*.^34^	Phase III, DB	Ukraine	BC	II/ III/ IV	202	0/202	Lipegfilgrastim vs. Pegfilgrastim	6 mg/cycle vs. 6 mg/cycle
24	Gladkov *et al*.^35^	Phase III, OL	Russian	BC	I/ II/ III/ IV	172	0/172	Balugrastim vs. Pegfilgrastim	40 mg/cycle vs. 6 mg/cycle
25	Volovat *et al*.^36^	Phase III, DB	Romania	BC	NA	381	0/381	Balugrastim vs. Pegfilgrastim	40 mg/cycle vs. 6 mg/cycle
26	Lee *et al*.^37^	Phase III, DB	SK	BC	NA	116	0/116	Pegteograstim vs. Pegfilgrastim	6 mg/cycle vs. 6 mg/cycle
27	Xu *et al*.^38^	Phase III, NA	China	BC/NSCLC	NA	500	61/439	Pegfilgrastim vs. Filgrastim	6 mg or 100 µg/kg/cycle vs. 5 µg/kg/day
28	Xie *et al*.^39^	Phase III, OL	China	BC	NA	569	5/564	Pegfilgrastim vs. Filgrastim	6 mg or 100 µg/kg/cycle vs. 5 µg/kg/day
29	Blackwell *et al*.^40^	Phase III, DB	USA	BC	I/ II/ III	214	0/214	S-G-CSF Bio vs. Filgrastim	5 µg/kg/day vs. 5 µg/kg/day
30	Park *et al*.^41^	Phase III, OL	SK	BC	I/ II/ III/ IV	74	0/74	L-G-CSF Bio vs. Filgrastim	6 mg/cycle vs.100 µg/m^2^/day
31	Park *et al*.^42^	Phase II, OL	SK	BC	II/ III	41	0/41	L-G-CSF Bio vs. Filgrastim	6 mg/cycle vs.100 µg/m^2^/day
32	Hegg *et al*.^43^	Phase III, OL	Brazil	BC	II/ III/ IV	217	0/217	S-G-CSF Bio vs. Filgrastim	5 mg/m^2^/day vs.5 mg/m^2^/day
33	Blackwell *et al*.^44^	Phase III, DB	USA	BC	I/ II/ III/ IV	308	0/308	L-G-CSF Bio vs. Pegfilgrastim	6 mg/cycle vs. 6 mg/cycle
34	Harbeck *et al*.^45^	NA, DB	Germany	BC	I/ II/ III/ IV	316	0/316	L-G-CSF Bio vs. Pegfilgrastim	6 mg/cycle vs. 6 mg/cycle
35	Waller *et al*.^46^	Phase III, DB	Germany	BC	NA	278	0/278	S-G-CSF Bio vs. Filgrastim	5 mg/kg/day vs. 5 µg/kg/day
36	Gatzemeier *et al*.^47^	Phase III, DB	Brazil	LC	Limited/Extensive	237	188/49	S-G-CSF Bio vs. Filgrastim	5 mg/kg/day vs. 5 µg/kg/day
37	A. Engert *et al*.^48^	Phase III, SB	Germany	NHL	NA	92	48/44	S-G-CSF Bio vs. Filgrastim	5 mg/kg/day vs. 5 µg/kg/day
38	Giglio *et al*.^49^	Phase III, SB	Brazil	BC	II/ III/ IV	348	2/346	S-G-CSF Bio vs. Filgrastim vs.Placebo	5 mg/kg/day vs. 5 µg/kg/day vs. -
39	Gisselbrecht *et al*.^50^	Phase III, DB	France	NHL	I/ II/ III/ IV	162	93/69	Lenograstim vs. Placebo	5 µg/kg/day. -
40	Bui *et al*.^51^	Phase II, DB	France	Sarcoma	Advanced	48	26/22	Lenograstim vs. Placebo	5 µg/kg/day vs. -
41	Nabholtz *et al*.^52^	Phase III, DB	USA	BC	II/ III/ IV	274	0/274	Leridistim vs. Filgrastim	5 µg/kg/day vs. 5 µg/kg/day
42	Welte *et al*.^53^	Phase III, OL	Germany	ALL	NA	34	27/7	Filgrastim vs. Placebo	5 µg/kg/day vs. -
43	Pettengell *et al*.^54^	NA, OL	UK	NHL	I/ II/ III/ IV	80	53/27	Filgrastim vs. Placebo	230 kg/m^2^/day vs. -
44	Johnston *et al*.^55^	NA, OL	USA	NSCLC	NA	13	8/5	Pegfilgrastim vs. Filgrastim	30/100/300 μg/kg/cycle vs. 5 μg/kg/day
45	Timmer-Bonte *et al*.^56^	Phase III, OL	Dutch	SCLC	I/ II/ III/ IV	175	113/62	Filgrastim vs. Placebo	300 μg/kg/cycle vs. -
46	Crawford *et al*.^57^	Phase III, DB	USA	SCLC	I/ II/ III/ IV	199	128/72	Filgrastim vs. Placebo	230 μg/m^2^/cycle vs. -
47	Osby *et al*. study1^58^	NA, NA	Sweden	Lymphoma	I/ II/ III/ IV	205	106/99	Filgrastim vs. Placebo	5 µg/kg/day vs. -
48	Osby *et al*. study2^58^	NA, NA	Sweden	Lymphoma	I/ II/ III/ IV	250	134/116	Filgrastim vs. Placebo	5 µg/kg/day vs. -
49	Trillet-Lenoir *et al*.^59^	Phase III, DB	France	SCLC	I/ II/ III/ IV	129	89/40	Filgrastim vs. Placebo	230 µg/m^2^/day vs. -
50	Zinzani *et al*.^60^	NA, NA	Italy	NHL	II/ III/ IV	149	69/80	Filgrastim vs. Placebo	5 mg/kg/day vs. -
51	von Minckwitz *et al*.^61^	NA, NA	Germany	BC	I/ II/ III/ IV	682	0/682	Pegfilgrastim vs. Filgrastim	6 mg/cycle vs. 5 µg/kg or 150 µg/m^2^/day
52	Balducci *et al*. study1^62^	Phase IV, OL	USA	LC/BC/OC	NA	686	235/451	Pegfilgrastim vs. Placebo	6 mg/cycle vs. -
53	Balducci *et al*. study2^62^	Phase IV, OL	USA	NHL	NA	146	69/77	Pegfilgrastim vs. Placebo	6 mg/cycle vs. -
54	Doorduijn *et al*.^63^	Phase III, NA	Dutch	NHL	II/ III/ IV	389	216/173	Filgrastim vs. Placebo	300 μg/day vs. -
55	Chevallier *et al*.^64^	Phase III, DB	France	BC	NA	120	0/120	Lenograstim vs. Placebo	5 µg/kg/day vs. -
56	Gebbia *et al*.^65^	NA, NA	Italy	BC/SCLC/HNC/HC/GC	Advanced	86	31/55	Filgrastim vs. Placebo	5 µg/kg/day vs. -
57	Romieu *et al*.^66^	Phase II, OL	France	BC	II/ III	60	0/65	Pegfilgrastim vs. Placebo	6 mg/cycle vs. -
58	Bozzoli *et al*.^67^	NA, NA	Italy	DLBCL	I/ II/ III/ IV	51	20/31	Pegfilgrastim vs. Filgrastim	6 mg/cycle vs. 300 μg/day
59	Filon *et al*.^68^	Phase III, DB	Russia	BC	II/ III/ IV	82	0/82	Empegfilgrastim vs. Filgrastim	6 mg/cycle vs. 5 μg/kg/day
60	Salafet *et al*.^69^	Phase II, OL	Russia	BC	NA	39	0/39	Empegfilgrastim vs. Filgrastim	6 mg/cycle vs. 5 μg/kg/day
61	Satheesh *et al*.^70^	NA, NA	India	BC	NA	71	0/71	Pegfilgrastim vs. Filgrastim	6 mg/cycle vs. 5 μg/kg/day
62	Glaspy *et al*.^71^	Phase II, OL	USA	BC	I/ II/ III/ IV	232	0/232	L-G-CSF Bio vs. Pegfilgrastim	80/240/320 µg/kg/cycle vs. 6 mg/cycle
63	Usuki *et al*.^72^	NA, NA	Japan	AML	NA	245	158/87	Filgrastim vs. Placebo	200 µg/m^2^/day vs. -
64	Desai *et al*.^73^	Phase III, DB	Canada	BC	II/ IIIB	589	0/589	L-G-CSF Bio vs. Pegfilgrastim	6 mg/cycle vs. 6 mg/cycle
65	Ottmann *et al*.^74^	Phase III, OL	Germany	ALL	NA	76	51/25	Filgrastim vs. Placebo	5 µg/kg/day vs. -
66	Bondarenko *et al*.^75^	Phase II, DB	Ukraine	BC	II/ III/ IV	104	0/104	Lipegfilgrastim vs. Pegfilgrastim	6 mg/cycle vs. 6 mg/cycle
67	Godwin *et al*.^76^	Phase III, DB	USA	AML	NA	211	122/89	Filgrastim vs. Placebo	400 µg/m^2^/day vs. -
68	Gladkov *et al*.^77^	Phase II, OL	Russian	BC	I/ II/ III/ IV	47	0/47	Balugrastim vs. Pegfilgrastim	40 mg/cycle vs. 6 mg/cycle
69	Michon *et al*.^78^	Phase II, OL	France	Neuroblastoma	IV	60	43/17	Filgrastim vs. Placebo	5 µg/kg/day vs. -
70	Maher *et al*.^79^	Phase III, DB	Australia	SC/ALL/Lymphoma	NA	216	103/113	Filgrastim vs. Placebo	12 μg/kg/day vs. -
71	Gatzemeier *et al*.^80^	Phase III, OL	Germany	SCLC	Limited/Extensive	280	231/49	Lenograstim vs. Placebo	150 µg/m^2^/day vs. -
72	Seymour *et al*.^81^	Phase I/II, SB	UK	SC/Lymphoma	NA	28	9/19	Lenograstim vs. Placebo	5 µg/kg/day vs. -
73	Muhonen *et al*.^82^	NA, NA	Finland	BC	IV	31	0/31	Filgrastim vs. Placebo	5 µg/kg/day vs. -

Note: NA, not available; M, male; F, female; SD, standard deviation; DB, double-blind; OL, open-label; SB, single-blind;

USA, the United States of America; UK, United Kingdom; SK, South Korea;

SCLC, Small-cell lung carcinoma; GCM, Germ cell malignancy; HL, Hodgkin lymphoma; ALL, Acute lymph oblastic leukemia; CRC, Colorectal cancer; BC, Breast cancer; NSCLC, Non-small-cell lung carcinoma; HNC, head and neck cancer; NHL, Non-Hodgkin lymphoma; AML, Acute myeloid leukaemia; LC, Lung cancer; OC, Ovarian cancer; HC, Hvarian cancer; GC, Gastric cancer; DLBCL, Diffuse large B-cell lymphoma; SC, Solid cancer;

Bio, Biosimilar.

**Figure 2:**
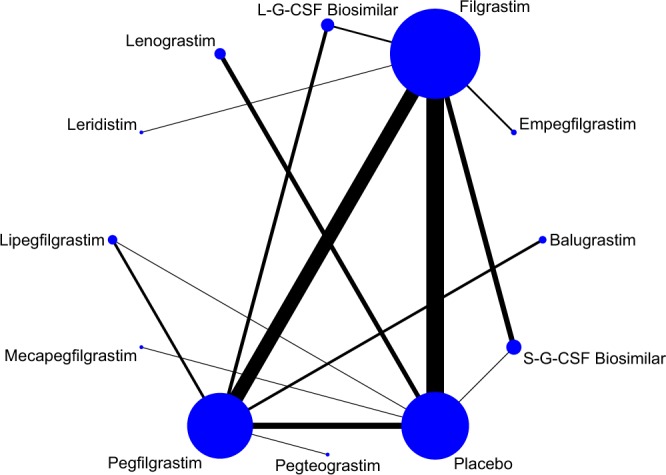
The network of the Bayesian network meta-analysis. Each node represents the treatment, and the size is proportional to the number of patients included. Each line represents the direct comparisons between treatments, and the width of the line is proportional to the number of randomized controlled trials.

Eligible comparisons for the multiple-treatments network meta-analysis were shown in Fig. 2. A total of 66 trials containing 13,770 patients were included in the FN analysis, a total of 41 trials containing 9,298 patients were included in the SN analysis, and a total of 45 trials containing 10,021 patients were included in the BP analysis. Furthermore, 72 RCTs were two-arm trials, while only one RCT was a three-arm trial, which compared S-G-CSF biosimilar, filgrastim and placebo. Moreover, 46 trials respectively contained more than 100 participants, and most of the participants were between 45 and 65 years old.

### Efficacy and tolerability of G-CSF drugs from pair-wise meta-analysis

A traditional direct pair-wise meta-analysis was performed, as shown in Table 2. The result revealed that filgrastim, pegfilgrastim, lenograstim and mecapegfilgrastim could reduce the incidence of FN (OR [95% CI]: 0.49 [0.38, 0.62]; 0.18 [0.06, 0.56]; 0.47 [0.29, 0.76]; 0.05 [0.00, 0.96]) and SN (OR [95% CI]: 0.29 [0.22, 0.38]; 0.16 [0.06, 0.47]; 0.37 [0.19, 0.72]; 0.30 [0.11, 0.81]) compared with placebo. Furthermore, the OR of mecapegfilgrastim compared with placebo was the lowest, but only one trial was included. The incidence of BP was greater in patients treated with filgrastim, pegfilgrastim, or lenograstim, when compared to placebo (OR [95% CI]: 2.07 [1.08, 3.97]; 1.91 [1.27, 2.87]; 8.31 [4.11, 16.80]). Filgrastim was better than leridistim in terms of reducing the incidence of FN (OR [95% CI]: 0.32 [0.11, 0.90]), but was worse than S-G-CSF biosimilar with regard to the incidence of BP (OR [95% CI]: 0.54 [0.30, 0.99]). Filgrastim was worse than pegfilgrastim in terms of reducing the incidence of FN (OR [95% CI]: 1.46 [1.07, 1.99]). The heterogeneity of these meta-analyses was mostly low or moderate. In the meta-analysis of RCTs that compared pegfilgrastim with placebo, a high heterogeneity was observed with FN (*I*^2^ = 89%), SN (*I*^2^ = 91%), and BP (*I*^2^ = 56%). This heterogeneity might have been introduced by the variation that resulted from the multiple types of tumors, since there were approximately five kinds of tumors in these seven trials. Since the sample size for every specific kind of tumor in these trials containing multiple types of tumors that was too small, it was difficult to implement an effective subgroup analysis. In the sensitivity analysis, no significant heterogeneity change was observed after removing studies from the analysis.

**Table 2:** Response for efficacy (FN and SN) and tolerability (BP) in the pair-wise meta-analysis.

	FN					SN					BP				
	Trial No.	Patients	Treatment (reponder/total)	OR [95% CI]	I^2^ (P value)	Trial No.	Patients	Treatment (reponder/total)	OR [95% CI]	I^2^ (P value)	Trial No.	Patients	Treatment (reponder/total)	OR [95% CI]	I^2^ (P value)
**Filgrastim vs**.															
Pegfilgrastim	16	3399	184/1547 vs. 155/1852	**1.46 [1.07, 1.99]**	8%(0.36)	12	2860	782/1265 vs. 948/1595	1.07 [0.90, 1.27]	0%(1.00)	11	1843	137/829 vs. 127/1014	1.40 [0.81, 2.40]	46%(0.05)
L-G-CSF Biosimilar	2	115	5/59 vs. 7/56	0.66 [0.17, 2.56]	9%(0.30)	0	0	—	—	—	2	116	6/59 vs. 8/57	0.65 [0.18, 2.37]	—
S-G-CSF Biosimilar	6	1371	63/627 vs. 61/744	1.04[0.59, 1.84]	35%(0.18)	3	681	202/300 vs. 266/381	0.94 [0.63, 1.41]	31%(0.24)	3	607	16/203 vs. 54/404	**0.54 [0.30, 0.99]**	0%(0.81)
Empegfilgrastim	2	121	1/59 vs. 2/62	0.64 [0.08, 5.41]	0%(0.60)	2	120	46/58 vs. 44/62	1.52 [0.53, 4.35]	31%(0.23)	0	0	—	—	—
Leridistim	1	910	5/135 vs. 15/139	**0.32 [0.11, 0.90]**	—	1	274	98/135 vs. 105/139	0.86 [0.50, 1.47]	—	0	0	—	—	—
Placebo	16	2460	300/1300 vs. 434/1160	**0.49 [0.38, 0.62]**	32%(0.11)	8	1409	307/701 vs. 474/708	**0.29 [0.22, 0.38]**	0%(0.47)	10	1673	81/739 vs. 45/739	**2.07 [1.08, 3.97]**	36%(0.12)
**Pegfilgrastim vs**.															
Balugrastim	3	517	8/260 vs. 7/257	1.07 [0.37, 3.12]	0%(0.63)	3	516	154/260 vs. 155/256	0.93 [0.62, 1.39]	14%(0.31)	3	598	30/262 vs. 43/336	0.87 [0.52, 1.46]	1%(0.36)
L-G-CSF Biosimilar	4	927	40/670 vs. 7/775	1.12 [0.71, 1.78]	0%(0.53)	1	227	35/65 vs. 95/162	0.82 [0.46, 1.47]	—	1	589	141/260 vs. 149/329	**1.43 [1.03, 1.98]**	—
Lipegfilgrastim	2	292	4/148 vs. 1/144	2.99 [0.46, 19.22]	0%(0.97)	2	292	77/148 vs. 60/144	1.52 [0.96, 2.41]	0%(0.49)	2	306	22/155 vs. 24/151	0.86 [0.46, 1.62]	0%(0.43)
Pegteograstim	1	115	9/59 vs. 11/56	0.74 [0.28, 1.94]	—	0	0	—	—	—	0	0	—	—	—
Placebo	7	3251	40/1627 vs. 269/1624	**0.18 [0.06, 0.56]**	89%(0.00)	6	2323	219/1164 vs. 620/1159	**0.16 [0.06, 0.47]**	91%(0.00)	6	3188	285/1595 vs. 197/1593	**1.91 [1.27, 2.87]**	56%(0.04)
**Lenograstim vs**.															
Placebo	3	330	93/165 vs. 119/165	**0.47 [0.29, 0.76]**	0%(0.41)	1	164	43/82 vs. 60/80	**0.37 [0.19, 0.72]**	—	5	633	65/318 vs. 10/315	**8.31 [4.11, 16.80]**	0%(0.70)
**Lipegfilgrastim vs**.															
Placebo	1	375	6/250vs. 7/125	0.41 [0.14, 1.26]	—	1	374	80/249vs. 74/125	**0.33 [0.21, 0.51]**	—	1	357	21/250 vs. 8/125	1.34 [0.58, 3.12]	—
**Mecapegfilgrastim vs**.															
Placebo	1	139	0/93vs. 4/46	**0.05 [0.00, 0.96]**	—	1	139	8/93vs. 11/46	**0.30 [0.11, 0.81]**	—	1	139	4/93 vs. 1/46	2.02 [0.22, 18.63]	—

Note: FN, febrile neutropenia; SN, severe neutropenia; BP, bone pain; OR, odds ratios; CI, confidence interval;

OR with statistical significance are in bold.

### Efficacy and tolerability of G-CSF drugs from network meta-analysis

**Figure 3:**
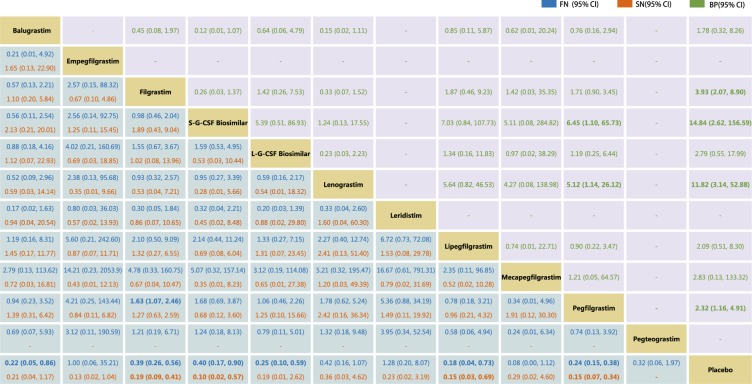
The pooled odds ratios (ORs) for the efficacy (FN and SN) and tolerability (BP) of the 12 treatments. The ORs are the column treatments compared with the row treatments in efficacy (FN and SN), and the row treatments compared with the column treatments in tolerability (BP). The results of efficacy (FN and SN) are in blue and orange, and the results of tolerability (BP) are in green. The first line of efficacy (FN and SN) in blue is the OR of FN, while the second line in orange is the OR of SN. The numbers in bold indicate the significant results. -, not compared.

Figure 3 summarizes the results of the random-effects network meta-analysis for the efficacy of G-CSF drugs based on FN and SN and acceptability, in terms of BP. There was no direct comparison trial of pegteograstim (transverse line indicate no comparison in Fig. 3) on SN, or direct comparison trial of empegfilgrastim, leridistim, and pegteograstim on BP. Pegfilgrastim significantly reduced the incidence of FN, when compared with filgrastim (OR [95% CI]: 1.63 [1.07–2.46]). There was no difference among other drugs in reducing the incidence of FN and SN. Compared with placebo, filgrastim, S-G-CSF biosimilar, lipegfilgrastim and pegfilgrastim significantly (*P* < 0.05) reduced the incidence of FN and SN, while balugrastim and L-G-CSF biosimilar reduced the incidence of SN. Although the difference was not statistically significant (95% CI contains 1), a reduction in the incidence of FN and SN was observed when empegfilgrastim, lenograstim, leridistim, mecapegfilgrastim, and pegteograstim were compared with placebo. The reason may be because the number of trials included was too small. In terms of the incidence of BP, S-G-CSF biosimilar and lenograstim significantly led to more than pegfilgrastim (OR [95% CI]: 6.45 [1.10–65.73]; 5.12 [1.14–26.12]). The incidence of BP by filgrastim, S-G-CSF biosimilar, lenograstim, and pegfilgrastim was significantly higher than placebo. However, there was no difference between other G-CSF drugs in the incidence of BP. By contrasting direct with indirect evidence using the node-split method, the network analysis did not reveal any statistical inconsistency with regards to FN, SN and BP.

### Comparison of the possibility of efficacy and tolerability of G-CSF drugs

**Figure 4:**
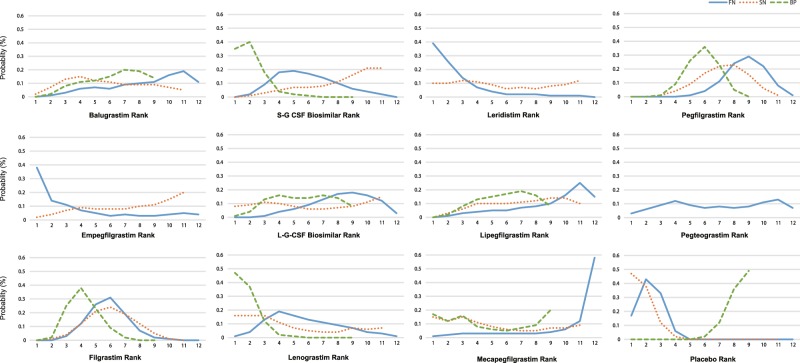
The ranking of treatments for efficacy (FN and SN) and tolerability (BP).

Figure 4 shows the distribution of possibility rank of the 12 treatments in terms of FN, SN, and BP. The higher the probability rank of the 12 treatment, the lower the probability of FN, SN and BP. Mecapegfilgrastim, lipegfilgrastim and balugrastim may be among the three best effective G-CSF drugs that could prevent the incidence of FN (cumulative probabilities: 58%, 15%, and 11%, respectively). S-G-CSF biosimilar, empegfilgrastim, and L-G-CSF biosimilar are possibly among the three more favorable G-CSF drugs that could prevent the occurrence of SN (cumulative probabilities: 21%, 20%, and 15%, respectively). Mecapegfilgrastim, balugrastim, lipegfilgrastim and L-G-CSF biosimilar were ranked as the lowest G-CSF drugs on incidence of BP (cumulative probabilities: 20%, 14%, 8%, and 8%, respectively).

## Discussion

In the present network meta-analysis, the efficacy and tolerability of 11 different G-CSF drugs for cancer patients after chemotherapy in 73 RCTs containing 15,124 patients were evaluated using FN, SN and BP as indicators. It was found that pegfilgrastim was better than filgrastim in reducing FN, and more tolerable than S-G-CSF biosimilar and lenograstim in terms of the incidence of BP. In terms of both efficacy and tolerance, mecapegfilgrastim, lipegfilgrastim and balugrastim might be the most efficacious and tolerable among G-CSF drugs.

Since FN is the main and severe adverse event for many chemotherapy regimens, and is intimately associated with chemotherapy-related mortality^83^, FN was chosen as the primary outcome of the G-CSF drug treatment and a crucial indicator to evaluate the efficacy of G-CSF drugs. In the present study, it was found that compared with placebo, most of the G-CSF drugs could reduce the risk of the incidence of FN, except for empegfilgrastim, leridistim, and pegteograstim. While leridistim might have an opposite effect, although the effect was not statistically significant. The network meta-analysis revealed that there was no difference or inferiority among the tested G-CSF drugs, except for filgrastim and pegfilgrastim in FN (filgrastim *vs*. pegfilgrastim OR [95% CI]: 1.63 [1.07–2.46]). Filgrastim, pegfilgrastim, lipegfilgrastim and lenograstim reduced the incidence of FN in cancer patients undergoing chemotherapy compared with placebo. Lipegfilgrastim appeared to lead to a greater reduction in the incidence of FN, when compared to pegfilgrastim and filgrastim, although the difference was not statistically significant. These findings were consistent with the previous observations^10,84^. In accordance with previous reports, pegfilgrastim was more effective than filgrastim in reducing the incidence of FN^10,85–88^. SN is also another important evaluation indicator of G-CSF drug efficacy. Filgrastim, pegfilgrastim, lipegfilgrastim, S-G-CSF biosimilar, mecapegfilgrastim, and lenograstim reduced the incidence of SN in patients undergoing myelosuppressive chemotherapy based on direct and indirect evidence. All these results indicate that compared with placebo, most of the tested G-CSF drugs were effective to prevent the incidence of FN and SN.

BP is one of the most common adverse events associated with G-CSF drug treatment^89^, and is an indicator of G-CSF drug tolerance. Filgrastim (OR [95% CI]: 3.93 [2.07, 8.90]), lenograstim (OR [95% CI]: 11.82 [3.14, 52.88]), pegfilgrastim (OR [95% CI]: 2.32 [1.16, 4.91]) and S-G-CSF biosimilar (OR [95% CI]: 14.84 [2.62, 156.59]) led to a higher incidence of BP, when compared with placebo. Lenograstim (OR [95% CI]: 5.12 [1.14, 26.12]) and S-G-CSF biosimilar (OR [95% CI]: 6.45 [1.10, 65.73)]) led to a much higher incidence of BP than pegfilgrastim. However, the level of incidence of BP widely varied among the RCTs of G-CSF drugs, which might have resulted from the differences in race of patients, stage and type of tumors, chemotherapy regimens, and definition of BP. These results suggest that patients might have different tolerances to different G-CSF drugs.

Even though there was no difference in efficacy among the tested G-CSF drugs and tolerability among patients to these G-CSF drugs in the pair-wise meta-analysis, the comparative ranking of these 12 G-CSF drug treatments suggest that mecapegfilgrastim, lipegfilgrastim and balugrastim might be more effective than leridistim, filgrastim and S-G-CSF biosimilar in preventing the incidence of FN, and S-G-CSF biosimilar, empegfilgrastim and L-G-CSF biosimilar might be more effective than filgrastim and pegfilgrastim in preventing the incidence of SN. In terms of BP, mecapegfilgrastim, balugrastim, lipegfilgrastim and L-G-CSF biosimilar might be more tolerable for patients, when compared to other G-CSF drugs. Those results indicate that mecapegfilgrastim, lipegfilgrastim and balugrastim might be the most efficacious and tolerable G-CSF drugs, and might provide a guideline for the selection of G-CSF-drugs for patients after chemotherapy.

Caution should be taken in interpreting the results, since there might be inconsistencies between the direct and indirect comparisons. These inconsistencies might have resulted from the different characteristics of trials, such as the study design, definition of indicators, inclusion criteria of subjects, and method of implementation, as well as the difference in identifying the external effect on the mean effect of the specific comparison between the network meta-analysis and pair-wise meta-analysis methods^90^. Although no inconsistency was found in FN, SN and BP through the node-split method in the main network analysis, the direct and indirect meta-analyses revealed contradictory results in terms of the comparisons between filgrastim *vs*. S-G-CSF biosimilar and filgrastim *vs*. L-G-CSF biosimilar. This mutually exclusive result could be explained as follows^90^: (1) if the direct evidence of the pair-wise meta-analysis was true, the comparison between other G-CSF drugs in indirect evidence of the network meta-analysis might overstate or understate the efficacy and tolerance; (2) if the indirect evidence was true, significant intrinsic heterogeneity might exist in the comparison among filgrastim, S-G-CSF biosimilar and L-G-CSF biosimilar. A low or moderate heterogeneity was observed in the pair-wise meta-analysis, indicating that the direct pair-wise meta-analysis was true.

Although the present study is the first network meta-analysis to comprehensively assess clinically and commonly used G-CSF drugs, it should be acknowledged that there were some limitations with the present analysis. First, many factors correlated with neutropenia after chemotherapy were not analyzed, such as the duration of neutropenia, duration of SN, depth of the absolute neutrophil count (ANC) nadir, time to recovery of ANC, FN-related hospitalization, and other toxic or side effects of G-CSF drugs. Second, in most of the included trials, the report for FN, SN and BP was incomplete, which caused some of the G-CSF drugs to be ruled out for comparison in terms of SN and BP. Third, trials on some G-CSF drugs were too few to be assessed. For example, merely one trial on mecapegfilgrastim has been reported to date. Fourth, the definition of BP and other indicators varied among these studies. Furthermore, the dose of G-CSF drugs also varied across the studies. These might be the source of heterogeneity and inconsistency. Fifth, the outcomes might only apply to developed countries, since some G-CSF drugs are not available on the market in many developing countries.

In summary, based on the present network meta-analysis, evidence suggests that compared with placebo, most of the tested G-CSF drugs are not different in terms of efficacy and tolerability, except for pegfilgrastim, which is more effective than filgrastim in reducing FN. Furthermore, pegfilgrastim is more tolerable for patients, when compared to S-G-CSF biosimilar and lenograstim, in terms of BP. Mecapegfilgrastim, lipegfilgrastim and balugrastim might be the most appreciate G-CSF drugs, which have both better efficacy and tolerance. It is noteworthy that more large-scale RCTs would be required to further confirm the efficacy and tolerance of the G-CSF drugs observed in the present study. The benefit-risk ratio of these G-CSF drugs still deserves to be further explored.

## Methods

### Search strategies and selection criteria

A network meta-analysis was performed following the PRISMA (preferred reporting items for systematic reviews and meta-analyses) guidelines^84^ and PRISMA network meta-analysis extension statement^91^. RCTs on 11 G-CSF drugs (balugrastim, empegfilgrastim, filgrastim, S-G-CSF Biosimilar, L-G-CSF Biosimilar, lenograstim, leridistim, lipegfilgrastim, mecapegfilgrastim, pegfilgrastim, and pegteograstim) for cancer patients after cytotoxic chemotherapy were searched in PubMed, Embase, Cochrane Library, Cochrane Collaboration Central Register of Controlled Clinical Trials, American Society of Clinical Oncology, and ClinicalTrials.gov up to the 8^th^ of October 2018, without language restrictions. The terms included “balugrastim”, “empegfilgrastim”, “filgrastim”, “Neupogen”, “G-CSF biosimilar”, “lenograstim”, “leridistim”, “lipegfilgrastim”, “mecapegfilgrastim”, “pegfilgrastim”, “Neulasta”, “pegteograstim”, “GCPGC”, “rhG-CSF”, “PEG-rhG-CSF” and “Pegylated Recombinant Human Granulocyte Colony Stimulating Factor” (Detailed terms can be found in supplementary appendix. S3). The reference lists of the relevant retrieved articles and reviews were also manually searched.

RCTs that compared at least two different G-CSF drugs (placebo-controlled included) in all kinds of cancer after chemotherapy were included. These trials should report the data on FN, SN, and/or BP in cancer patients after the use of G-CSF drugs. Non-randomized controlled trials, non-interventional studies, retrospective studies, or trials that contained only one treatment (single-arm) were excluded. Furthermore, studies that included healthy volunteers, but not cancer patients who received chemotherapy, were also excluded.

### Study selection and data extraction

Study selection, data extraction and review, and quality assessment were independently performed by two authors (Y. Wang and L. Chen), according to the predefined criteria from eligible studies. The Cochrane Collaboration’s tool for assessing risk of bias^92^ was independently used for the quality assessment and evaluation of risk of bias by the same authors. The key characteristics of each study were recorded, which included: the first author’s name and year of publication, country, study design, patient characteristics, chemotherapy regimens, dose and protocol of treatment, and outcomes (FN, SN and BP). All data for the study characteristics and clinical responses were summarized in a structured table to ensure consistency. All the disagreements were resolved by discussion and consensus with a third author (Y. Li).

### Outcome measurements

The incidence of FN after cytotoxic chemotherapy within two weeks was taken as the primary indicator of efficacy of G-CSF drugs, the incidence of SN was taken as the secondary indicator of G-CSF drug efficacy, and BP was taken as the primary indicator for the tolerability of G-CSF drugs. FN was defined as an absolute neutrophil count (ANC) of <0.5 or 1.0 × 10^9^/L, with an oral temperature of ≥38.0 °C. SN was defined as ANC < 0.5 or 1.0 × 10^9^/L. If both data of both grade 3 and 4 bone marrow suppression (ANC < 0.5 and 1.0 × 10^9^/L) were reported in a study, the data of the ANC < 0.5 × 10^9^/L was used with priority for analysis, because grade 3 had lesser clinical significance, and was not always reported in the included studies.

### Statistical analyses

Pair-wise meta-analysis was carried out for FN, SN and BP to compare the corresponding interventions. The random effects model for pair-wise meta-analysis was used to account for the heterogeneity. The heterogeneity among different trials was estimated by Cochran’s Q-test (*P* < 0.05 indicated significant heterogeneity) and *I*^2^ statistic. If *I*^2^ = 0–25%, it is designated as low heterogeneity, if *I*^2^ = 25–50%, this was designated as moderate heterogeneity, if *I*^2^ = 50–75%, this was designated as high heterogeneity, and if *I*^2^ = 75–100%, this was designated as extremely high heterogeneity. According to the Cochrane handbook, heterogeneity can be accepted when *I*^2^ ≤ 50%^93^. Pair-wise meta-analysis was performed using Review Manager 5.3 (Cochrane Collaboration, Oxford, UK) and STATA 12.0 (Stata Corporation, TX, USA) statistical software.

Random-effects models were applied for the network meta-analysis. Bayesian network meta-analysis was used to combine the collected data. The Bayesian network meta-analysis was performed with WinBUGS version 1.4.3 (MRC Biostatistics Unit, Cambridge, UK). Random effects models were used to incorporate the effects from different studies, while heterogeneity within the comparison was evaluated in a relatively conservative and appropriate manner^94^. The models were performed using the Markov Chain Monte Carlo simulation. The initial values were set for three different chains, 150,000 interactions with 5,000 burn-in samples were produced to obtain the model parameters from the posterior distributions, and 50 thinning rates were adopted for each chain. The odds ratios (ORs) were collected or calculated from combing the direct evidence, and the significance was assessed by *P* < 0.05, or the 95% confidence interval (CI) did not contain 1. The best efficacious and tolerant regimen was confirmed by ranking the included G-CSF drugs according to the OR for each G-CSF drug compared with placebo, and assessing the probability. Inconsistencies in the present study were assessed by comparing the direct evidence with indirect evidence from the network meta-analysis using the node-split method^95^.

A sensitivity analysis was performed by determining whether there was statistically significant heterogeneity in the meta-analysis after studies were randomly removed from the others.

## Supplementary information

Supplementary
