# Supplementary material for: Efficacy and tolerability of granulocyte colony-stimulating factors in cancer patients after chemotherapy: A systematic review and Bayesian network meta-analysis
Source: Sci Rep. 2019 Oct 25;9:15374. doi: 10.1038/s41598-019-51982-4 (PMC6814815; doi:10.1038/s41598-019-51982-4)
Supplement: Supplementary file 1 — Supplementary [file 41598_2019_51982_MOESM1_ESM.pdf]

Efficacy and tolerability of granulocyte colony-stimulating factors in cancer patients after chemotherapy: A systematic review and Bayesian network meta-analysis

Yong Wang[1,2], Lin Chen[3], Fen Liu[4], Ning Zhao[4], Liyao Xu[5], Biqi Fu[6], Yong Li[1*]

Authors' Affiliations:

[1]Department of Medical Oncology, The First Affiliated Hospital of Nanchang University, 17 Yongwai Zheng Road, Nanchang 330000, China;

[2]Department of Medical Oncology, The Affiliated Ganzhou Hospital of Nanchang University (Ganzhou People's Hospital), 18 Meiguan Road, Ganzhou 341000, China;

[3]Department of Internal Neurology, The Affiliated Ganzhou Hospital of Nanchang University (Ganzhou People's Hospital), 18 Meiguan Road, Ganzhou 341000, China;

[4]Critical Care Medicine, The First Affiliated Hospital of Nanchang University, 17 Yongwai Zheng Road, Nanchang 330000, China;

[5]Department of paediatrics, Children's Hospital, Zhejiang University School of Medicine, 57 Zugan Road, Hangzhou, 310000, China;

[6]Department of Rheumatology, The First Affiliated Hospital of Nanchang University, 17 Yongwai Zheng Road, Nanchang 330000, China.

***Corresponding Author:** Dr. Yong Li, Department of Medical Oncology, The First Affiliated Hospital of Nanchang University, 17 Yongwai Zheng Road, Nanchang 330000, China. Tel.: +86-15879155066.

E-mail: liyongcsco@email.ncu.edu.cn

Supplementary Figure. S1 Risk of bias graph

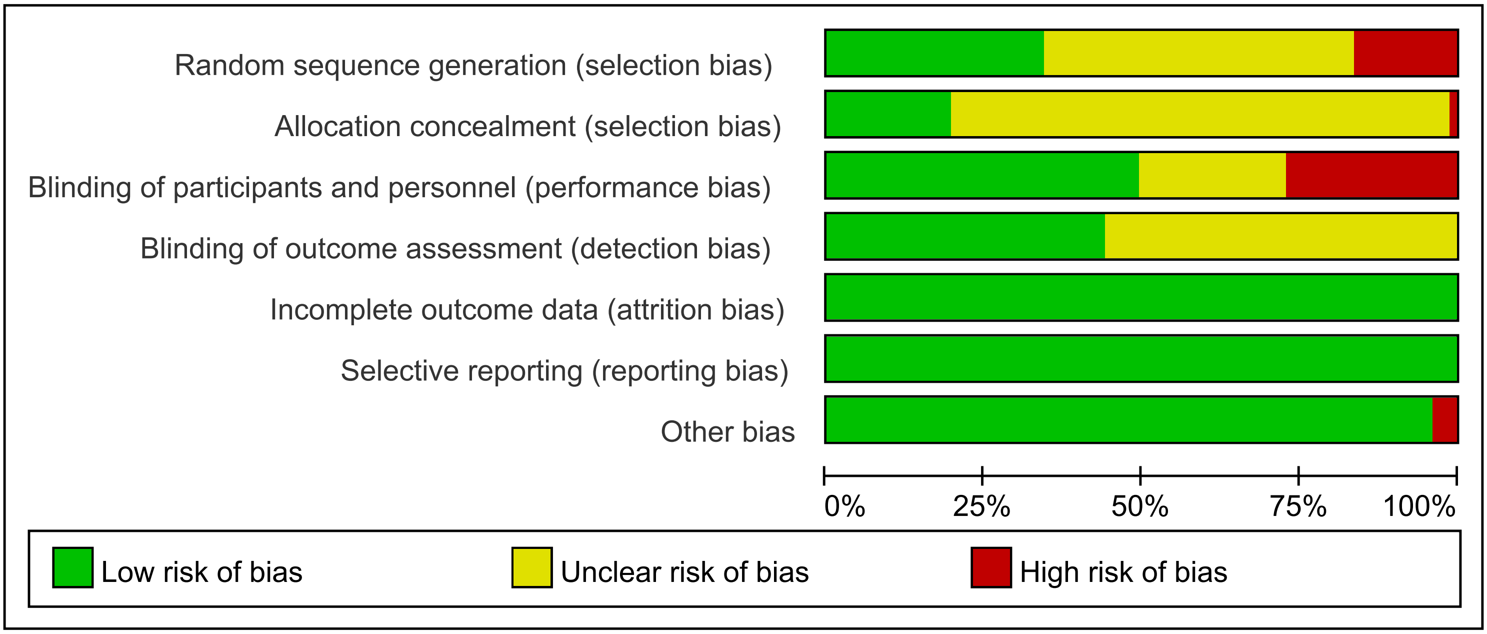

Supplementary Figure. S2 Risk of bias summary

	Random sequence generation (selection bias)	Allocation concealment (selection bias)	Blinding of participants and personnel (performance bias)	Blinding of outcome assessment (detection bias)	Incomplete outcome data (attrition bias)	Selective reporting (reporting bias)	Other bias
A. Engert et al. 2009	?	+	−	+	+	+	+
Balducci et al. 2007 study1	+	+	+	+	+	+	+
Balducci et al. 2007 study2	+	+	+	+	+	+	+
Blackwell et al. 2015	+	+	+	+	+	+	+
Blackwell et al. 2016	?	+	+	+	+	+	+
Bondarenko et al. 2013	?	+	+	+	+	+	+
Bondarenko et al. 2015	+	+	+	+	+	+	+
Bozzoli et al. 2015	?	?	?	?	+	+	+
Buchner et al. 2014	?	−	+	+	+	+	+
Bui et al. 1995	?	?	+	+	+	+	+
Chevallier et al. 1995	?	?	+	+	+	+	+
Crawford et al. 1991	−	?	+	+	+	+	+
Crawford et al. 2005	−	?	+	+	+	+	+
Desai et al. 2018	?	?	+	?	+	+	+
Doorduijn et al. 2003	?	?	?	?	+	+	+
Dunlop et al. 1998 study1	+	?	?	?	+	+	+
Dunlop et al. 1998 study2	+	?	?	?	+	+	+
Filon et al. 2015	+	?	+	+	+	+	+
Fosså et al. 1998	?	?	?	?	+	+	+
Fox et al. 2009	+	?	+	+	+	+	+
Gatzemeier et al. 2000	?	?	−	?	+	+	+
Gatzemeier et al. 2009	?	?	+	+	+	+	+
Gebbia et al. 1993	?	?	?	?	+	+	+
Geissler K et al. 1997	?	?	?	?	+	+	+
Giglio et al. 2008	?	+	−	+	+	+	+
Gisselbrecht et al. 1997	+	?	+	+	+	+	+
Gladkov et al. 2015	?	?	−	?	+	+	+
Gladkov et al. 2016	+	+	+	?	+	+	+
Glaspy et al. 2014	+	?	−	?	+	+	+
Godwin et al. 1998	−	?	+	+	+	+	+
Green et al. 2003	+	+	+	?	+	+	+
Grigg et al. 2003	?	?	−	?	+	+	+
Harbeck et al. 2016	−	?	+	+	+	+	+
Hecht et al. 2010	−	?	+	+	+	+	+
Hegg et al. 2016	?	?	−	?	+	+	+
Holmes et al. 2002 study1	?	?	+	+	+	+	+
Holmes et al. 2002 study2	?	?	+	+	+	+	+
Johnston et al. 2000	?	?	−	?	+	+	+
Kosaka et al. 2015	+	+	+	?	+	+	+
Kubo et al. 2016	+	?	+	?	+	+	+
Lee et al. 2016	?	?	+	+	+	+	+
Maher et al. 1991	+	+	+	+	+	+	+
Michon et al. 1998	+	?	−	?	+	+	+
Muhonen et al. 1996	?	?	?	?	+	+	+
Nabholtz et al. 2002	−	+	+	+	+	+	+
Osby et al. 2003 study1	?	?	?	?	+	+	+
Osby et al. 2003 study2	?	?	?	?	+	+	+
Ottmann et al. 1995	?	?	−	?	+	+	+
Park et al. 2013	+	?	−	?	+	+	+
Park et al. 2017	−	?	−	?	+	+	+
Pettengell et al. 1992	+	?	−	?	+	+	+
Pinter et al. 2017	−	?	+	+	+	+	+
Romieu et al. 2007	−	?	−	?	+	+	+
Salafet et al. 2013	+	?	−	?	+	+	+
Satheesh et al. 2009	?	?	?	?	+	+	+
Seymour et al. 1995	?	?	?	?	+	+	+
Shi et al. 2013	+	?	−	?	+	+	−
Sierra et al. 2008	+	+	+	+	+	+	+
Timmer-Bonte et al. 2005	?	?	−	?	+	+	+
Trillet-Lenoir et al. 1993	?	?	+	+	+	+	+
Usuki et al. 2002	−	?	?	?	+	+	+
Vogel et al. 2005	−	?	+	+	+	+	+
Volovat et al. 2014	−	?	+	+	+	+	+
Volovat et al. 2015	+	?	+	+	+	+	+
von Minckwitz et al. 2008	?	?	?	?	+	+	+
Vose et al. 2003	?	?	−	?	+	+	+
Waller et al. 2010	+	?	+	+	+	+	+
Welte et al. 1996	?	?	−	?	+	+	+
Xie et al. 2018	?	?	−	?	+	+	+
Xu et al. 2016	?	?	?	?	+	+	−
Zhang et al. 2015	+	?	?	?	+	+	+
Zhou et al. 2016	+	?	+	?	+	+	−
Zinzani et al. 1997	?	?	?	?	+	+	+

Supplementary appendix. S3 Detailed terms for search strategies

Detailed terms for search strategies

((((((((((((((filgrastim[Title/Abstract]) OR Neupogen[Title/Abstract]) OR rhG-CSF[Title/Abstract]) OR pegfilgrastim[Title/Abstract]) OR Neulasta[Title/Abstract]) OR Mecapegfilgrastim[Title/Abstract]) OR Lipegfilgrastim[Title/Abstract]) OR lonquex[Title/Abstract]) OR Pegteograstim[Title/Abstract]) OR GCPGC[Title/Abstract]) OR PEG-rhG-CSF[Title/Abstract]) OR Pegylated Recombinant Human Granulocyte Colony Stimulating Factor[Title/Abstract]) OR G-CSF biosimilar[Title/Abstract]) OR lenograstim[Title/Abstract])
